# Enhancing psychological well-being of school teachers in India: role of energy management, thriving, and stress

**DOI:** 10.3389/fpsyg.2023.1239587

**Published:** 2023-10-20

**Authors:** Smita Chaudhry, Raina Chhajer

**Affiliations:** ^1^Department of Human Resources, FLAME School of Business, FLAME University, Pune, India; ^2^Humanities and Social Sciences, Indian Institute of Management Indore, Indore, India

**Keywords:** psychological well-being, energy management, thriving, stress, school teachers, India

## Abstract

**Purpose:**

The psychological well-being of school teachers is a growing concern in the post-pandemic era. Many initiatives are undertaken by individual school authorities and government agencies to address this issue. In this study, we examine the impact of energy management, thriving, and stress on the psychological well-being of school teachers in India.

**Method:**

Data was collected from 356 school teachers in Rajasthan, India, through a cross-sectional questionnaire survey. These teachers were working both in rural and urban areas. The relationship among energy management, thriving, stress, and psychological well-being was analyzed using hierarchical regression.

**Results:**

The findings suggest that energy management positively impacts psychological well-being. A mediating effect of thriving and stress on the relationship between energy management and psychological well-being was also found. The results support that psychological well-being can be enhanced by investing in managing school teachers’ energy levels.

**Discussion:**

These results contribute to our theoretical and practical understanding of factors that can enhance the psychological well-being of school teachers and improve the quality of education. Institutes may design and implement interventions on energy management to enhance the psychological well-being of school teachers.

## Introduction

School teachers deal with daily classroom and student management challenges (Fenwick, 1998). They have to manage the learning environment, pedagogy with different types of students within the given learning environment, their own identity, and informal student interactions. This impacts their energy levels, resulting in emotional fatigue and lethargy. Research indicates that these factors lead to burnout among school teachers, leading to intentions to leave the profession (Blix et al., 1994). Many of them cannot cope with work stress, leading to stress-related health problems. They also have lower psychological well-being due to significantly lesser perceived control regarding autonomy, authenticity, connection to others, and resilience than other professionals (Grenville-Cleave and Boniwell, 2012).

School teachers’ psychological well-being has especially been impacted during the Covid-19 pandemic, as they had to manage multiple tasks. Research conducted during the pandemic revealed that uncertainty, workload, negative perception about the job, concern for others’ well-being, health struggles, and playing multiple roles hampered school teachers’ mental health and well-being (Kim et al., 2022). The sudden introduction of distance learning, including adopting new technology, revising pedagogy, and mode of assessment to deliver online education efficiently, was a challenge for many school teachers in India (Bhattacharya and Tandon, 2023; Dayal, 2023).

Psychological well-being implies having a sense of purpose, personal growth, environmental mastery, self-acceptance, autonomy in thoughts and actions, and positive interpersonal relationships (Ryff and Singer, 2006; Ryff and Singer, 2008). School teachers need to maintain their psychological well-being not only for their health, but also to be effective educators. Glazzard and Rose (2020) argue that school teachers’ psychological well-being is essential for the quality of education they provide. It can impact students’ motivation, engagement, and academic achievement, thus affecting their performance and well-being. Psychological well-being enables teachers to sustain their teaching performance and make a desirable impact on their pupils (Rahm and Heise, 2019; Greenier et al., 2021).

Energy management can be a valuable and essential tool to enhance psychological well-being. Energy management refers to managing one’s physical, emotional, mental, and spiritual energy levels to maintain a state of optimal functioning (Ryan and Frederick, 1997). School teachers dedicate long hours to preparing interactive sessions, conducting in-class lectures, grading assignments, attending to their students’ ongoing learning challenges, and involving students in diverse activities for their overall development. Energy as a limited resource gets exhausted in attending to these various demands. Teachers proficient in managing their energy are better equipped to handle the demands and challenges of their profession. Research indicates that energy management promotes work engagement and job satisfaction while reducing burnout (Parker et al., 2017).

**Figure 1:**
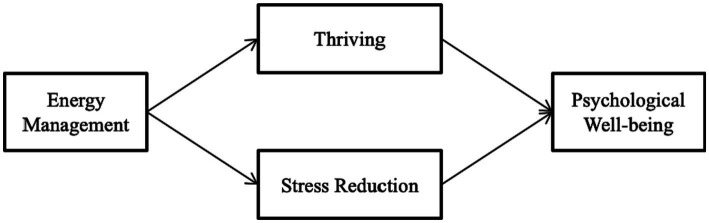
Guiding model.

Energy management practices implemented by school teachers might impact their psychological well-being. This study examines the impact of energy management on school teachers’ psychological well-being. Our study also explores the role of thriving and stress in determining the impact (see Figure 1). Thriving implies having an experience of both vitality and learning at the same time (Carmeli and Spreitzer, 2009). Stress is defined as a negative emotional and physiological response to challenging events or situations (Lazarus and Folkman, 1984). Theory indicates energy management as an antecedent of thriving, yet empirical research is required to explain this relationship. Further, the relationship between energy management, thriving, stress, and psychological well-being remains unexplored in the context of Indian school teachers.

Although the study is based in the Indian context, it can be helpful to any other country for two reasons, as indicated by the literature. First, school teachers may experience poor psychological well-being due to factors that may be applicable anywhere in the world like work–family conflict (Zhou et al., 2021), life and work circumstances (Jelińska and Paradowski, 2021) and challenges of the teaching occupation (Fenwick, 1998; Grenville-Cleave and Boniwell, 2012). Second, teachers strongly influence their students by virtue of their profession. Their low well-being can hamper the well-being and learning of the students (Roeser et al., 2022; Wu and Lu, 2022). Thus, the study is vital in any geographical context to understand the measures that can be taken to enhance teachers’ psychological well-being.

The remainder of this paper is organized as follows. First, we review the existing literature on psychological well-being, energy management, thriving, and stress and hypothesize the relationship between the variables. Second, we describe our research method to collect and analyze data to test the hypothesized relationships. Third, we present our results based on the analysis, including descriptive statistics and regression analyses. Fourth, we discuss the implications of our findings for theory and practice, recommendations for improving school teachers’ psychological well-being, and areas for future research. Finally, we conclude with a summary of our study. In the following section, we explore the literature on psychological well-being, energy management, thriving, and stress and the theoretical basis of hypothesizing the relationship among them.

## Theory and hypothesis

### Psychological well being

Well-being refers to a state of optimal functioning characterized by the presence of positive emotions, a relative absence of negative emotions, and satisfaction with one’s life (Keyes, 2007). It is a crucial aspect of an individual’s overall health and quality of life. Psychological well-being is a fundamental aspect of human life and encompasses various dimensions of an individual’s psychological functioning (Mehrotra et al., 2013). Ryff and Singer (2006, 2008) identified six primary dimensions of psychological well-being: a sense of purpose, personal growth, environmental mastery, self-acceptance, autonomy in thoughts and actions, and positive interpersonal relationships. Individuals with higher levels of psychological well-being tend to have a greater sense of purpose in life, are open to personal growth, exhibit confidence in influencing their environment, accept themselves for who they are, maintain high-quality relationships with others, and think and act independently.

Keyes (2013) stated that psychological well-being promotes self-awareness, satisfaction of emotional needs, attainment of intrinsic goals, self-reliance, and the ability to act with consciousness. It involves developing personal resources, positive relationships, purposeful engagement in life activities, and self-realization (Keyes, 2002). Psychological well-being is a dynamic process that reflects individuals’ ability to adapt to life’s challenges and maintain a positive outlook toward their future (Mehrotra et al., 2013).

Psychological well-being has been found to be a crucial factor in promoting work engagement among teachers. Studies have shown that teachers with higher levels of psychological well-being have higher work engagement, which is defined as the level of enthusiasm, involvement, and dedication toward work (Greenier et al., 2021). Studies have also shown that teachers suffering from depression can adversely affect students. Wu and Lu (2022) found that teachers’ depression can negatively affect students’ academic achievement and emotional well-being. Thus, schools and educational institutions must prioritize teachers’ psychological well-being to ensure positive outcomes for teachers and students.

The recent COVID-19 pandemic has had a significant impact on the well-being of teachers, resulting in depression, anxiety, and burnout (Kotowski et al., 2022; Lacomba-Trejo et al., 2022; Lizana and Lera, 2022; Ramos et al., 2022). Such ill effects have been widely reported in recent studies, highlighting the importance of understanding and addressing these issues to support the mental health of educators.

### Energy management

According to job demand-resources theory (JD-R), individuals must maintain specific personal resources at work to combat everyday job demands (Bakker and Demerouti, 2017). This equilibrium helps in sustaining individuals’ engagement levels and performance at work. One such personal resource that may impact the psychological well-being of individuals is their levels of physical, emotional, mental, and spiritual energy.

Energy management refers to effectively using and replenishing one’s personal resources to achieve desired outcomes (Ryan and Frederick, 1997). In the context of teachers, it refers to efficiently using their physical, emotional, and cognitive resources to fulfill their work responsibilities.

Teachers in India face various challenges that impact their energy levels and ability to perform their duties effectively. The teaching profession is known for its demanding nature, which can often lead to burnout, hampering their energy levels. It has been found that teacher burnout is a significant problem in India (Shukla and Trivedi, 2008). Teachers who are fatigued, stressed, or burnt out may be less effective in delivering instruction and engaging with students. According to Shukla and Trivedi (2008), demographic factors such as age, gender, and marital status have also been found to impact the energy level of teachers.

Energy management can have a significant impact on the well-being of individuals. Sugiura et al. (2005) conducted a study to examine the relationship between physical activity and psychological well-being. They found that physical activity levels were significantly related to psychological well-being, with individuals who engaged in more physical activity reporting higher levels of psychological well-being. Research on school teachers has found that physical activity promotes mental health (Aperribai et al., 2020). Low physical activity is associated with low well-being, stress, depression, and anxiety in teachers (Çifçi and Demir, 2020; Biernat et al., 2022).

A study conducted by Parker et al. (2017) examined the momentary effects of different types of work-related energy management strategies on employee well-being. The researchers hypothesized that energy management is part of a resource investment process to maintain and improve well-being. They found that the effectiveness of this resource investment process depended on the type of strategy used and the existing drain on resources, such as job demands. The study’s findings suggested that adaptive energy management strategies that promote effective resource investment can help individuals maintain and improve their well-being. The study also showed that prosocial energy management, which involves using personal energy to help others, was positively related to job satisfaction. This suggests that energy management practices involving social support and connection can positively impact individual well-being.

Thus, teachers proficient in managing their energy would experience higher levels of psychological well-being.

*H1*: Energy management is positively related to psychological well-being.

### Thriving

Thriving is a psychological state in which individuals experience vitality (an affective dimension) and learning (a cognitive dimension) (Spreitzer et al., 2005). Vitality is the experience of having energy and feeling alive at work. Learning refers to a sense of greater understanding, knowledge, and skills at work. Thriving individuals have a feeling of energy and continuous growth and development at work. Both vitality and learning are essential for thriving. They might feel depleted and burnt out if they are learning but lack energy. Alternatively, if they are energized but are not learning, they might stagnate at work. Thriving reflects an individual’s ability to flourish and reach their full potential (Spreitzer et al., 2005). It increases job satisfaction, engagement, and performance (Saks, 2006).

Literature suggests that energy management may enable thriving in the workplace (Spreitzer et al., 2005). Energy management involves regulating and optimizing personal energy levels to enhance performance, while thriving involves flourishing in multiple domains of life, including work. Different energy management strategies, including emotional, mental, and physical, are positively associated with work engagement (Zhang et al., 2022), which is related to thriving (Saks, 2006). Studies have also indicated that energy management enhances positive affect, a source for thriving at work (Spreitzer et al., 2005). Proper exercise and movement (cardiovascular and strength training), nutrition (a balanced combination of carbohydrates, fats, and proteins), and sleep (seven to eight hours per night) increase positive moods during the workday (Spreitzer and Grant, 2012). Emotional energy management has been found to be effective in activating recovery processes (Zhang et al., 2022). Individuals adopt strategies like energy audits to sustain their energy levels (Spreitzer and Grant, 2012).

The concept of thriving is closely related to psychological well-being and has been found to impact individual outcomes favorably. Teachers who reported higher levels of thriving reported lower levels of stress and burnout (Shukla and Trivedi, 2008). The vitality dimension of thriving pertains to high energy and enthusiasm for work, which indicates positivity toward work. A study of teachers found that high levels of both vitality and learning (i.e., high thriving) were associated with more favorable outcomes related to satisfaction and burnout. In contrast, low vitality/high learning extremes were associated with a wide range of less desirable outcomes, such as emotional exhaustion and depersonalization (Keith, 2021).

Therefore, we propose that teachers proficient in managing their energy are more likely to experience a thriving state, leading to better psychological well-being.

*H2*: Thriving mediates the relationship between energy management and psychological well-being.

### Stress

Stress is a complex physiological and psychological response to perceived or actual threats, challenges, or demands that disrupt individuals’ homeostasis and require them to adapt or cope with the situation (Selye, 1976; Lazarus and Folkman, 1984). The experience of stress is influenced by various individual and environmental factors, including personal characteristics, coping strategies, social support, and job demands (Lazarus and Folkman, 1984). Stress can positively and negatively affect individuals’ health, well-being, and performance, depending on the type, intensity, and duration of stressors and their response to them (Cohen et al., 1997; McEwen, 2000).

Energy management is associated with lower stress. According to job demand and resource theory, if job demands exceed job resources, individuals tend to experience stress (Bakker and Demerouti, 2017). For example, high job demands, such as workload or job complexity, can lead to high-stress levels and energy depletion. Conversely, job resources such as social support, feedback, and job control can lower stress levels. At the same time, the conservation of resources theory posits that individuals are motivated to acquire, maintain, and protect their resources (Hobfoll, 1989). According to the theory, stress occurs when individuals perceive that their resources are threatened or depleted. Considering that energy is a personal resource, the two theories imply that individuals who conserve or manage their energy levels (vis-à-vis job demands) tend to experience lower stress (Hobfoll and Freedy, 1993).

Stress and psychological well-being have been studied extensively as related constructs, with research findings suggesting that stress can negatively impact psychological well-being (Sugiura et al., 2005). Individuals who experience higher levels of stress are more likely to have lower levels of psychological well-being, including lower levels of positive affect, life satisfaction, and self-esteem. Segrin et al. (2007) found that stress plays a mediating role in the relationship between social skills and different aspects of psychological well-being. Individuals with better social skills tend to experience less stress, contributing to higher psychological well-being. They also found that stress is linked to higher levels of depression and anxiety and reduced life satisfaction.

Therefore, we propose that teachers proficient in managing their energy are less likely to experience stress, leading to better psychological well-being.

*H3*: Stress mediates the relationship between energy management and psychological well-being.

The following section explains the research methodology for testing the hypotheses. We elaborate on the data collection procedure, demographics of the sample, measures for the variables, and verification of data validity and reliability.

## Method

### Sample and procedure

An online survey was conducted during May 2022 in the state of Rajasthan, India. Six hundred fifty school teachers were invited to participate in our study using a questionnaire-based survey from various schools. Participants were recruited via convenience sampling. School teachers who were employed in public or private schools for more than a year, who completed at least their graduation, were included in the study. Three hundred fifty-six school teachers responded after giving their informed consent, yielding a response rate of 54.77%. No significant difference was found between respondents and non-respondents in terms of their gender, location of school (urban/rural), type of school (private/public), and method of teaching (online/offline). Thus, there is no evidence of non-response bias. We found ten missing values across nine responses that we imputed using the mode or median (depending on the repetition of values) of the concerned variable for the specific respondent. This imputation method was suitable since the missing values were less than 5% (Schafer, 1997).

Participants were acquainted with the objectives and procedures of the survey, and anonymity and confidentiality were assured. The procedure involved asking individual school teachers to rate their energy level, thriving, stress, and psychological well-being using a self-reported online survey questionnaire. On completion of the survey, we shared an information sheet with school authorities and requested them to circulate the same with their school teachers. The information sheet included details of different sources of energy, various energy management practices, and the energy audit tool designed by Spreitzer and Grant (2012). The Institutional Review Board of the Indian Institute of Management Indore approved the study procedure (reference number IRB/01/2022-23/HSS).

The study sample consisted of 206 female respondents (57.9%) and 146 male respondents (41%), with 42.7% of the responses received from urban schools and 57.3% from rural schools. 253 responses were from public schools, and the remaining were from private schools. Most respondents (58.7%) had over ten years of work experience, and the average age of the participants was 36.65 years.

The sample had a significant number of respondents (58.1%) who worked in higher secondary schools, and most (81.2%) used offline teaching modes. Many of the respondents (73.3%) had a post-graduate degree. Regarding personal information, most respondents were married (87.1%), with children distributed across different age groups. Additionally, 48% of the respondents had no support system at home, such as parents, in-laws, or maids, while 72.2% had caregiving responsibilities. Detailed demographic information about the respondents is presented in Table 1.

**Table 1:** Demographic profile of school teachers.

S. no.	Demographic variables	Statistics
1	Gender	Male: 41% Female: 58% Prefer not to say: 0.8%
2	Employment Type	Public schools: 71.1% Private schools: 28.9%
3	School Type	Primary: 20.8% Middle: 15.4% Secondary: 5.6% Higher Secondary: 58.1%
4	School Location	Urban: 42.7% Rural: 57.3%
5	Teaching Mode	Offline: 81.2% Online: 2.5% Hybrid: 16.3%
6	Work Experience	Less than 2 years: 3.4% 2 to 5 years: 18.5% 5 to 10 years: 19.4% More than 10 years: 58.7%
7	Education	Graduate: 23.9% Ph.D.: 2.8% Post Graduate: 73.3%
8	Daily time dedicated to teaching preparation	Less than 2 h: 29.2% 2 to 5 h: 42.7% 5 to 8 h: 23.3% More than 8 h: 4.8%
9	Number of students in class	Less than 20 students: 9.8% 20 to 40 students: 11.0% 40 to 60 students: 30.1% More than 60 students: 15.4% Not mentioned: 33.7%
10	Marital Status	Married: 87.1% Single: 9.6% Divorced: 0.6% Widow: 2.8%
11	Number of children	Zero: 16.9% One: 21.6% Two: 56.2% Three: 5.3%;
12	Age of the youngest child	Less than one year: 3.7% Between 1 and 5 years: 14.6% Between 6 and 10 years: 10.4% Between 11 and 15 years: 14.3% More than 15 years: 41.3% Not mentioned: 15.7%
13	Having support at home	None: 52.0% Parents: 24.4% In-laws: 13.2% House helps: 10.4%
14	Caregiving responsibilities at home	None: 27.8% Child: 30.3% Old or ill senior citizen: 41.9%

*N* = 356.

The study employed Statistical Package for Social Sciences (SPSS version 28) by IBM Corporation to analyze the data using descriptive statistics, exploratory factor analysis (EFA), and hierarchical regression. Initially, we subjected each variable to EFA using principal component analysis to identify and remove items with factor loadings less than 0.5 or significant cross-loadings. We then checked the reliability of the variables concerning the remaining items to identify variables with a Cronbach Alpha (α) reliability coefficient of less than 0.70 (Nunnally, 1994). Table 2 provides all variables’ factor loadings and reliability measures with their corresponding selected items.

**Table 2:** Item scales.

S. No.	Item scales	Factor loading
A	Energy management (*α* = 0. 87)	
1	I do not regularly get at least seven or eight hours of sleep, and I often wake up feeling tired.	0.59
2	I frequently skip breakfast, or I settle for something that is not nutritious.	0.57
3	I do not work out enough (meaning cardiovascular training at least three times a week and strength training at least once a week).	0.62
4	I do not take regular breaks during the day to truly renew and recharge, or I often eat lunch at my desk, if I eat at all.	0.65
5	I frequently find myself feeling irritable or anxious at work, especially when work is demanding.	0.68
6	I do not have enough time with my family and loved ones, and when I’m with them, I’m not always really with them.	0.68
7	I have too little time for the activities that I most deeply enjoy.	0.61
8	I do not stop frequently enough to express my appreciation to others or to savor my accomplishments and blessings.	0.56
9	I have difficulty focusing on one thing at a time, and I am easily distracted during the day, especially by email.	0.66
10	I do not take enough time for reflection, strategizing and creative thinking.	0.64
11	I do not spend enough time at work doing what I do best and enjoy most.	0.63
12	My decisions at work are more often influenced by external demands than by a strong, clear sense of my own purpose.	0.60
13	I do not invest enough time and energy in making a positive difference to others or to the world.	0.55
B	Thriving (*α* = 0.90)	
1	I find myself learning often	0.69
2	I continue to learn more and more as time goes by	0.72
3	I see myself continually improving	0.67
4	I have developed a lot as a person	0.77
5	I feel alive and vital	0.79
6	I have energy and spirit	0.78
7	I feel alert and awake	0.84
8	I am looking forward to each new day	0.81
C	Stress (*α* = 0.79)	
1	I feel upset because of something that happens unexpectedly	0.62
2	I feel that I am unable to control the important things in my life	0.77
3	I feel nervous and “stressed”	0.77
4	I find that I cannot cope with all the things that I have to do	0.60
5	I get angry because of things that are outside of my control	0.69
6	I feel difficulties are piling up so high that I cannot overcome them	0.72
D	Psychological well-Being (*α* = 0.84)	
1	I like most parts of my personality.	0.66
2	When I look at the story of my life, I am pleased with how things have turned out so far.	0.67
3	Some people wander aimlessly through life, but I am not one of them.	0.61
4	I am good at managing the responsibilities of daily life.	0.73
5	I sometimes feel as if I’ve done all there is to do in life.	0.55
6	For me, life has been a continuous process of learning, changing, and growth.	0.77
7	I think it is important to have new experiences that challenge how I think about myself and the world.	0.65
8	People would describe me as a giving person, willing to share my time with others.	0.56
9	I have confidence in my own opinions, even if they are different from the way other people think.	0.63
10	I judge myself by what I think is important, not by the values of what others think is important.	0.58

Cronbach Alpha = α; *N* = 356.

### Measures

#### Energy management

We adopted a 16-item energy management measure developed by Schwartz and Catherine (2007). Respondents had to answer using a seven-point Likert scale with the categories 1 = strongly disagree to 7 = strongly agree. Sample items include “I frequently find myself feeling irritable or anxious at work, especially when work is demanding.” After exploratory factor analysis, 13 items were retained, and three were dropped. The reliability of the energy management measure was 0.87 (Cronbach’s α).

#### Thriving

We adopted a 10-item thriving measure developed by Porath et al. (2012). Respondents had to provide answers using a seven-point Likert scale with the categories 1 = strongly disagree to 7 = strongly agree. Sample items include “I see myself continually improving.” After exploratory factor analysis, eight items were retained, and two were dropped. The reliability of the thriving measure was 0.90 (Cronbach’s α).

#### Stress

We adopted a 10-item perceived stress measure developed by Cohen et al. (1983). Respondents had to answer using a seven-point Likert scale with the categories 1 = strongly disagree to 7 = strongly agree. Sample items include “I find that I cannot cope with all the things that I have to do.” After exploratory factor analysis, six items were retained, and four were dropped. The reliability of the perceived stress measure was 0.79 (Cronbach’s α).

#### Psychological well-being

We adopted an 18-item perceived stress measure developed by Ryff and Keyes (1995). Respondents had to answer using a seven-point Likert scale with the categories 1 = strongly disagree to 7 = strongly agree. Sample items include “I like most parts of my personality.” After exploratory factor analysis, ten items were retained, and eight were dropped. The reliability of the perceived stress measure was 0.84 (Cronbach’s α).

In this study, age and gender were used as control variables.

In the following section, we present the quantitative analysis of the sample data and the results of hypotheses testing based on hierarchical regression in IBM SPSS v28.

## Results

We prepared the correlation matrix to better understand the responses regarding the critical variables under study: gender, age, energy management, thriving, stress, and psychological well-being (Table 3). By presenting the means, standard deviation, and correlation among these variables, the matrix helped comprehend the response patterns for the variables and the nature and significance of the relationship between them.

**Table 3:** Means, standard deviation, and inter-correlations.

	Variables	*M*	S.D.	1	2	3	4	5	6
1	Gender	0.60	0.51	1					
2	Age	36.65	7.00	−0.05	1				
3	Energy Management	4.35	1.26	−0.02	−0.02	1			
4	Thriving	5.98	0.92	−0.07	0.02	0.18**	1		
5	Stress	2.46	0.80	0.19**	0.00	−0.20**	−0.27**	1	
6	Psychological well being	5.78	0.83	−0.06	−0.02	0.26**	0.47**	−0.27**	1

*N* = 356. *p < 0.05; **p < 0.01; ***p < 0.001. Pearson correlation coefficient. Two-tailed significance values are reported.

The results for energy management showed a significant positive correlation with thriving (*r* = 0.18, *p* < 0.01), a negative correlation with stress (*r* = −0.20, *p* < 0.01), and a positive correlation with psychological well-being (*r* = 0.26, *p* < 0.01). The results for thriving showed a significant positive correlation with psychological well-being (*r* = 0.47, *p* < 0.01). The results for stress showed significant negative correlation with psychological well-being (*r* = −0.27, *p* < 0.01). The results indicate that high energy management is associated with thriving and psychological well-being. High stress is associated with low energy management and less psychological well-being. The relationship between the variables was consistent with the theoretical underpinnings, previous empirical findings, and our proposed hypotheses.

### Hypotheses testing

We tested the hypotheses using hierarchical regression of energy management, thriving, and stress on psychological well-being as a dependent variable (Table 4), and regression of energy management on thriving and stress as dependent variables. We used the parameters of standardized coefficients (*β*), *R*^2^, Δ*R*^2^, and Adjusted *R*^2^. *R*^2^ and Adjusted *R*^2^ indicate the proportion of the variance in the criterion variable explained by predictor variables. However, Adjusted *R*^2^ also considers the significance of the predictor variable in explaining the variance, i.e., it increases with the inclusion of a predictor variable only if it significantly impacts the criterion variable. The following section details the analysis of the hypotheses.

**Table 4:** Hierarchical regression for psychological well-being.

Predictor variables	Model 1	Model 2	Model 3A	Model 3B	Model 3C
Step 1 (Control variables)					
Gender	−0.07 (0.21)	−0.06 (0.24)	−0.03 (0.50)	−0.02 (0.70)	−0.01 (0.84)
Age	−0.05 (0.35)	−0.04 (0.48)	−0.03 (0.53)	−0.05 (0.31)	−0.04 (0.41)
Step 2 (Independent variable)					
Energy management		0.25*** (<0.001)	0.18*** (<0.001)	0.21*** (<0.001)	0.15** (<0.001)
Intervening Variables					
Step 3A					
Thriving			0.43*** (<0.001)		
Step 3B					
Stress				−0.23*** (<0.001)	
Step 3C					
Thriving					0.40*** (<0.001)
Stress					−0.14** (0.005)
*R*^2^	0.01	0.07	0.25	0.12	0.27
∆*R*^2^	0.01	0.07*** (<0.001)	0.18*** (<0.001)	0.05*** (<0.001)	0.20*** (<0.001)
Adjusted *R*^2^	0.00	0.06	0.24	0.11	0.26
*F*	0.83	8.79*** (<0.001)	29.19*** (<0.001)	11.8*** (<0.001)	25.32*** (<0.001)
∆F	0.83	24.58*** (<0.001)	84.17*** (<0.001)	19.46*** (<0.001)	46.70*** (<0.001)

*N* = 356. *p < 0.05; **p < 0.01; ***p < 0.001; All coefficients are standardized; One-tail significance values are reported. Model 1: Model with Gender and Age; Model 2: Model with Gender, Age, and Energy Management; Model 3A: Model with Gender, Age, Energy Management, and Thriving as a mediator; Model 3B: Model with Gender, Age, Energy Management, and Stress as a mediator; Model 3C: Model with Gender, Age, Energy Management, both Thriving and Stress as mediators.

#### Effect of energy management

Hypothesis 1 predicted that energy management is positively related to psychological well-being. Model 2 indicated a significant positive relation between the two variables (*β* = 0.25, *p < 0.*001), with energy management explaining an additional 7% of the variance in psychological well-being (Δ*R*^2^ = 0.07, *p* < 0.001; Adjusted *R*^2^ = 0.06), thus providing support for H1.

To test the mediation Hypotheses, we created three alternate models: Model 3A, Model 3B, and Model 3C. Model 3A specified the intervening role of only thriving. Model 3B specified the intervening role of only stress. Model 3C specified the intervening roles of both thriving and stress.

#### Thriving as a mediator

Hypothesis 2 predicted that thriving mediates the relation between energy management and psychological well-being, as energy management enables thriving that, in turn, increases psychological well-being. Regression of energy management on thriving indicated a significant positive relation (*β* = 0.18, *p < 0.*001), and Model 3A indicated a positive relation of thriving with psychological well-being (*β* = 0.43, *p < 0.*001). Also, the inclusion of thriving reduced the effect of energy management on psychological well-being (*β* = 0.18, *p < 0.*001) and explained an additional 18% of the variance in psychological well-being (Δ*R*^2^ = 0.18, *p* < 0.001; Adjusted *R*^2^ = 0.24). These results provide support for H2 on the mediation of thriving, according to the mediation method by Baron and Kenny (1986).

#### Stress as a mediator

Hypothesis 3 predicted that stress mediates the relationship between energy management and psychological well-being, as energy management reduces stress, which, in turn, decreases psychological well-being. Regression of energy management on stress indicated a significant negative relation (*β* = −0.20, *p < 0.*001), and Model 3B indicated the negative relation of stress with psychological well-being (*β* = −0.23, *p < 0.*001). Also, the inclusion of stress reduced the effect of energy management on psychological well-being (*β* = 0.21, *p < 0.*001) and explained an additional 5% of the variance in psychological well-being (Δ*R*^2^ = 0.05, *p* < 0.001; Adjusted *R*^2^ = 0.11). These results provide support for H3 on the mediation of stress, according to the mediation method by Baron and Kenny (1986).

A comparison between the three models revealed that thriving and stress together explained variance in psychological well-being the most, as shown in Model 3C (Δ*R*^2^ = 0.20, *p* < 0.001; Adjusted *R*^2^ = 0.26). When considered together, both thriving (*β* = 0.40, *p < 0.*001) and stress (*β* = −0.14, *p < 0.*001) mediated the relation between energy management and psychological well-being.

According to Cohen (2013), effect size, in terms of standardized coefficient (*β*), is small if it is between 0.10 and 0.29 and medium if it is between 0.30 and 0.49. The findings showed that the effect size was small for the relation of energy management with thriving, stress, and psychological well-being and the relation of stress with psychological well-being. The effect size was medium for the relation of thriving with psychological well-being.

Based on the study’s results, we discuss the theoretical and practical implications and future research avenues in the following section.

## Discussion

This paper explores the relationship between energy management, thriving, stress, and psychological well-being among school teachers in India. Education is considered the backbone of any country and plays a crucial role in shaping the nation’s future. Teachers are pivotal in delivering quality education and preparing students for the future. However, teaching is a demanding profession that can significantly impact teachers’ psychological well-being, especially in the post-pandemic era (Kim et al., 2022). Glazzard and Rose (2020) argue that teacher well-being is essential for the individual teacher’s mental and physical health and the quality of education they provide. It can impact students’ motivation, engagement, and academic achievement, thus affecting their performance and well-being. Our findings show that energy management can effectively enhance psychological well-being, by increasing a sense of thriving and reducing stress. This has implications for theory and practice.

The psychological well-being of teachers and its determinants have been extensively discussed in the literature. Studies have explored the role of several factors in determining psychological well-being, including acceptance of technology (Fülöp et al., 2022), professional identity (Zhao et al., 2022), affective, normative and continuance commitment (McInerney et al., 2015), growth mindset (Nalipay et al., 2022), coping strategies (Gustems-Carnicer and Calderón, 2013), social support and work autonomy (Kim et al., 2022), teacher efficacy (for example, in digital technologies) and emotional support (Chan et al., 2021), assessment periods, the pressure of extracurricular activities, the experience of the unexpected, keeping up with the pace of change and changes in school leadership (Glazzard and Rose, 2020), the ratio of positive to negative emotions (Rusu and Colomeischi, 2020), and perceived school administration values and their congruence with personal values (Wang and Hall, 2019).

However, limited literature associating energy management with school teachers’ psychological well-being exists. Few relevant studies have mainly focused on the dimension of physical energy (Aperribai et al., 2020; Çifçi and Demir, 2020; Biernat et al., 2022). Other education studies have discussed students’ energy management (Spreitzer and Grant, 2012). By highlighting the positive impact of physical, emotional, spiritual, and mental energy on the psychological well-being of school teachers, the study extends the literature on antecedents of psychological well-being in the education context (McInerney et al., 2015; Fülöp et al., 2022; Zhao et al., 2022).

Extant literature exists on antecedents of thriving (Prem et al., 2017; Rego et al., 2021; Babalola et al., 2022). In the context of school teachers, a few studies have explored the effect of a supportive work environment (Collie and Perry, 2019) and inter-collegial strengths (Moore et al., 2022) on thriving. However, the impact of energy management on thriving has yet to be examined in the context of school teachers. Besides, limited literature exists on the association of school teachers’ thriving with aspects related to well-being. Studies have examined the effect of thriving on burnout (Keith, 2021) and well-being (Okros and Virga, 2022). By investigating the role of school teachers’ thriving in the relationship between their energy management and psychological well-being, this study extends the existing literature on thriving in general and in the education context, particularly (Babalola et al., 2022; Moore et al., 2022).

Considerable studies have been conducted on stress factors (Schaubroeck et al., 1989; Universari and Harsono, 2021). In the context of school teachers, studies have discussed factors like principled leadership (Collie and Perry, 2019), educational technology (Fernández-Batanero et al., 2021), inadequate salary, and low status (Litt and Turk, 1985). However, the relation of stress with energy management has not been examined. Besides, studies have explored the negative impact of stress on teacher commitment to continue teaching, the formation of strong student-teacher relationships (Klassen et al., 2013), and the quality of online instructions (Panisoara et al., 2020). However, none of them have explored the impact of stress on well-being. By investigating the role of school teachers’ stress in the relationship between their energy management and psychological well-being, this study extends the existing literature on stress in general and stress in the education context in particular (Fernández-Batanero et al., 2021; Universari and Harsono, 2021).

### Practical implications

The findings have practical implications for both individual school authorities and government agencies. These institutions can come up with specific employee or process-level interventions that help develop and manage the physical, emotional, spiritual, and mental energy of the teachers. They can take the initiative to make the teachers more aware of the need to manage their energy and guide them in achieving this objective. These institutions can hold informative programs on physical, emotional, spiritual, and mental well-being for the teachers. These discussions could be held during weekly staff meetings, information on different energy sources could be disseminated via online or print materials, and an introduction to well-being could be included as a specific module during the teacher training program.

Institutions can conduct yoga, mindfulness, time management, and stress management workshops. These workshops could be conducted regularly online or in person during school hours. An hour-long session conducted thrice a week may include a sequence of whole-body stretching, breathwork, meditation, and a few well-being practices like writing gratitude letters, savoring, and strength spotting activities. Certified instructors may be invited to lead these programs. They can also offer active mental and emotional health support by appointing counselors or accessing online counseling services. These initiatives would help teachers build resilience and cope with the job demands.

Institutions can provide resources and take measures that help reduce the teachers’ workload, enable them to manage their classes and students better and create opportunities for learning, socializing, and building a support system within the peer group. Such initiatives would help the teachers conserve energy, develop professionally, and feel emotionally and socially connected to others. Moreover, teachers may also experience more positive affect, enthusiasm, satisfaction, and engagement in the institution, which would help them cope with daily challenges and manage their energy better. Similarly, government agencies can tie up with experts in the domains of energy management and psychological well-being to provide training to teachers across educational institutions in a systematic and organized manner.

### Limitations and future research avenues

Despite the theoretical and empirical contribution of the studies, the paper also has a limitation. We acknowledge that in this study, only self-reported measures were used that might have introduced potential response biases. Due to the self-reported data collected from a single source, the findings might have a possibility of common method bias. Besides, collection of cross-sectional data prevented us from analyzing causal relationships. Additionally, due to time constraints, we could collect data from only one state in India, which limits the generalizability of findings. To address this limitation, future research may include other states in India to measure cultural and contextual variations among school teachers across India.

Future research studies may include an experimental design to test this theoretical model further. An energy management workshop focused on enhancing teachers’ physical, emotional, spiritual, and mental energy could be a significant intervention to be tested. School teachers could be recruited to join energy management workshops by forming partnerships with state or district education offices. Forming such partnerships might further help in collecting data from a larger sample. School teachers may be randomly assigned to experimental and wait-list control groups. Data could be collected pre- and post-workshop and follow-up after one month. The findings might further investigate the causal relationship among the study variables and the longitudinal impact of the intervention. Examining the school teachers’ energy management practices and their psychological well-being over an extended period might provide further insights into the long-term effectiveness of interventions and their sustainability.

Lastly, studies can explore the role of other variables like family and institutional support, work engagement, and personal values in determining energy management’s effect on school teachers’ psychological well-being. For example, the Job-Demand and Resources Model of work engagement (Bakker and Demerouti, 2017; Cho et al., 2020) states that perceived support or personal values could be essential antecedents to work engagement, leading to enhanced psychological well-being. Using this theoretical framework, further research questions could be explored. The next section summarizes and highlights the salient aspects of the study.

## Conclusion

In India, education has been given considerable emphasis, with several policies being implemented to promote and enhance the quality of education. One of the factors that is imperative for this purpose is the psychological well-being of teachers who impart this education, especially in the post-pandemic times. Limited literature has discussed what actions can be taken at the individual level to increase psychological well-being. Our paper investigates whether psychological well-being can be increased if teachers have physical, emotional, spiritual, and mental energy. Data collected from 356 school teachers from the most significant state of India reveal that these dimensions of energy management can increase psychological well-being as they enhance thriving and reduce teachers’ stress. These results contribute to our theoretical and practical understanding of factors that can enhance the psychological well-being of school teachers and improve the quality of education in the long term.

## Data availability statement

The original contributions presented in the study are included in the article/supplementary material, further inquiries can be directed to the corresponding author.

## Ethics statement

The studies involving humans were approved by Indian Institute of Management Indore. The studies were conducted in accordance with the local legislation and institutional requirements. The participants provided their written informed consent to participate in this study.

## Author contributions

SC: research design, introduction, literature review, analysis, interpretation, and discussion. RC: conception of the study, variables, literature review, formation of hypotheses, data collection, and interpretation. All authors contributed to the article and approved the submitted version.
